# Spherical neutron polarimetry under high pressure for a multiferroic delafossite ferrite

**DOI:** 10.1038/s41467-018-06737-6

**Published:** 2018-10-22

**Authors:** Noriki Terada, Navid Qureshi, Laurent C. Chapon, Toyotaka Osakabe

**Affiliations:** 1 National Institute for Materials Science, Sengen 1-2-1, Tsukuba, Ibaraki 305-0047 Japan; 2 Institut Laue Langevin, CS 20156, 38042 Grenoble Cedex 9, France; 3 Japan Atomic Energy Agency, Tokai, Ibaraki 319-1195 Japan; 4 Diamond Light Source, Diamond House, Harwell Science and Innovation Campus, Didcot, Oxfordshire OX11 0DE UK

## Abstract

The analysis of three-dimensional neutron spin polarization vectors, using a technique referred to as spherical neutron polarimetry (SNP), is a very powerful means of determining complex magnetic structures in magnetic materials. However, the requirement to maintain neutrons in a highly polarized state has made it difficult to use this technique in conjunction with extreme experimental conditions. We have developed a high pressure cell made completely of nonmagnetic materials and having no effect on neutron polarizations. Herein, we report the first SNP analyses under high pressure up to 4.0 GPa in the magnetoelectric multiferroic delafossite CuFeO_2_. This study also determined the complex spiral magnetic structures in these pressure-induced phases, by measuring the full neutron polarization matrix. The results presented herein demonstrate that the SNP measurements are feasible under high pressure conditions, and that this method is a useful approach to study pressure-induced physical phenomena.

## Abstract (web summary)

Spherical neutron polarimetry (SNP) experiment in conjunction with high pressure condition faces many experimental difficulties. Here the authors report development of SNP with nonmagnetic hybrid anvil high pressure cell and application to the magnetoelectric multiferroic delafossite CuFeO2 system.

## Introduction

Since the first experimental demonstration of neutron polarization analysis^1^, this technique has been widely applied to various systems, including ferromagnets, superconductors, and multiferroics, in experiments involving diffraction^2–4^, inelastic scattering^5–7^, small-angle scattering^8,9^ and reflectometry^10,11^. Most of the polarized neutron experiments belong to longitudinal polarization analysis, in which neutron spins are analyzed along a single quantization axis. More recently, Tasset et al. developed the CRYOgenic polarization analysis device (CRYOPAD), which enables three dimensional neutron polarization analysis, or so-called spherical neutron polarimetry (SNP)^12,13^. The SNP technique makes it possible to determine precise spin orientations in complex magnetic structures. In unpolarized neutron diffraction experiments, one needs to collect many magnetic Bragg peaks obtained in different sample positions to perform the standard refinement procedure. In this case, the precision of the refined parameters, such as spin orientation, is affected by each intensity data accuracy including absorption and extinction corrections for different diffraction geometries. In contrast, in the SNP analysis, the sample position and diffraction geometry are identical for the matrix elements of each magnetic reflection, and the absorption and extinction effects cancel out as the ratio of spin-up and spin-down neutrons is measured. Therefore, we obtained the better precision of magnetic structure parameters even in the case of a small number of observable reflections.

However, since the CRYOPAD requires zero-magnetic field conditions in conjunction with superconducting Meissner screening to avoid neutron depolarization^12,13^, it is necessary to use equipment, such as high-pressure cells, inside the magnetic vacuum that are made of nonmagnetic materials. Other disadvantages associated with high-pressure SNP experiments are the lower incident neutron intensity compared to unpolarized neutron techniques, the limited sample volume, and the large absorption of the cell. In fact, to date, SNP experiments under high pressures have not been carried out owing to these difficulties. In the present study, we used a newly developed nonmagnetic Hybrid anvil high-pressure cell (HAC) to overcome the difficulties associated with performing SNP experiments under pressure. The HAC was originally developed by Osakabe for neutron diffraction experiments, and is able to generate hydrostatic pressure up to 10 GPa by using two different materials, WC (with a ferromagnetic Co binder) and sapphire (or silicon carbide, SiC), as anvils^14,15^. In order to use the HAC for SNP experiments, we employed a combination of a sapphire single crystal and a nonmagnetic diamond composite (with a SiC binder) or WC with a nonmagnetic Ni binder as the anvil materials. Nonmagnetic materials were also carefully selected for the other parts of the cell by assessing the magnetism of potential cell materials via low-temperature magnetization measurements (Supplementary Figure 1).

**Fig. 1:**
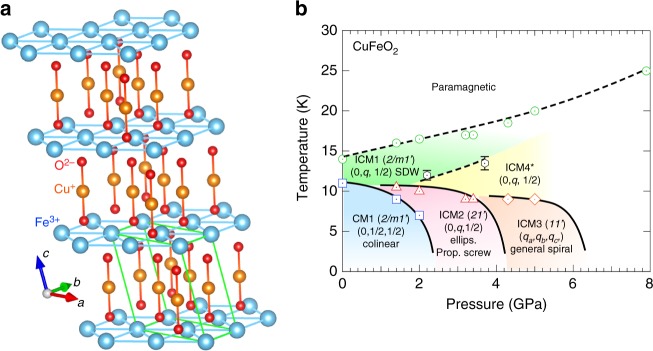
Crystal structure and magnetic and dielectric phase diagram as functions of temperature and pressure. **a** The crystal structure of delafossite CuFeO_2_, showing low-temperature monoclinic unit vectors. **b** A pressure versus temperature magnetic phase diagram for CuFeO_2_. Circle, squire, triangle, and diamond symbols were taken from the previous paper^17^. The phase transition points shown by hexagon symbols have been defined in the present work. The solid and dotted lines denote first and second order phase transitions, respectively. In the present experiment, it has not been clear that the ICM4 phase either exists as a single phase or coexists with ICM1 phase. Details are described in the main text

We selected the magnetoelectric (ME) multiferroic compound delafossite CuFeO_2_ for the first SNP experiment under high pressure. Since the multiferroic ferrite is expected to have various types of magnetic orderings under high pressure, such as collinear spin-density-wave (SDW), noncollinear spiral structures^16,17^, we considered that it was the best candidate to study the feasibility of the SNP analysis under pressure. ME multiferroics, possessing both (anti)ferromagnetic and ferroelectric orderings, have attracted much attention over the past decade with respect to both pure physics and potential practical applications^18–20^. Recently, it has been reported that the application of hydrostatic pressure or uniaxial stress can be used to tune the ferroelectric properties of these materials by changing their magnetic ordering/symmetry^21–28^. CuFeO_2_ has a triangular crystal structure with the space group R$\bar 3$m at room temperature, but a monoclinic structure with space group C2/m below its magnetic ordering temperature of 14 K (Fig. 1a)^29–32^. At ambient pressure, this compound undergoes successive magnetic phase transitions with decreasing temperature, at 14 and 11 K^33–36^, as illustrated in the pressure versus temperature phase diagram in Fig. 1b. The SDW ordering in this material, with collinear spins tilted by 18° from the hexagonal *c*-axis toward^1–10^ direction (monoclinic *a*-direction) and the incommensurate propagation vector, *k* = (0, *q*, 1/2; *q* ≃ 0.4), denoted as the ICM1 phase, is stabilized over the range of 11 K ≤ *T* ≤ 14 K^37^. Below 11 K, the commensurate *k* = (0, 1/2, 1/2) collinear magnetic structure with spins pointing the hexagonal *c*-axis (CM1 phase) appears as the ground state. These magnetic phases at ambient pressure have the nonpolar magnetic point group 2/m1′ (that is, paraelectric). In previous work, a ferroelectric polarization can be induced by application of magnetic field^38^ and chemical substitutions^39–43^ in CuFeO_2_.

The application of hydrostatic pressure to CuFeO_2_ also induces nonpolar to polar magnetic phase transitions from the CM1 phase to ICM2 phase (*k* = (0, *q*, 1/2; *q* ≃ 0.4)) at ∼3.0 GPa, followed by another polar phase, ICM3, (*k* = (*q*_a_, *q*_b_, *q*_c_; *q*_a_ ≃ 0, *q*_b_ ≃ 0.34, *q*_c_ ≃ 0.42)) at ∼4.0 GPa, as reported in previous study with unpolarized neutron diffraction^17^. However, the detailed parameters of the magnetic structure in ICM2 phase, such as the ellipsoidicity ratio, have not yet been determined. Moreover, due to a lack of sufficient observable Bragg reflections, a magnetic structural analysis of the ICM3 phase having the general k-vector could not be carried out in the previous study^17^. In addition, the stability of the SDW structure for the ICM1 phase at low temperature above 6 GPa has not yet been fully understood. Since SNP analysis can precisely determine the magnetic structural shape, even when only a small number of observable Bragg reflections are available, we anticipated that the detailed magnetic structures in ICM2 and ICM3 phases, and the low-temperature stability of the ICM1 phase can be investigated using the SNP technique. In this study, we develop the completely nonmagnetic HAC and successfully determine the magnetic structures in the pressure-induced phases in CuFeO_2_ by using the SNP experiments under high pressure.

## Results

### Ambient pressure phases

First, to test the feasibility of performing SNP experiments under pressure, we measured the full neutron polarization matrix, **P**_*αβ*_, at close to ambient pressure (0.2 GPa) using the HAC. In the present experiments, the single crystal was mounted with the *a*-axis vertical to provide access to the (0, *K*, *L*) reflections. **P**_*αβ*_ is defined to be a polarization ratio of scattered neutrons along *β*( = *x,y,z*) direction when incident neutron vector is parallel to *α*( = *x,y,z*) direction. *x*-axis is parallel to scattering vector, *z*-axis is perpendicular to the scattering plane, and *y*-axis is orthogonal to *x*-axis and *z*-axis in right-handed Cartesian coordination. Assuming an ellipsoidal spiral magnetic structure with a spiral axis along a general direction (of which collinear and helical structures are special cases), we can express the **P**_*αβ*_ matrix in Eq. 1 as described in Supplementary Note 2.

1 $$	{{\mathbf{P}}_{\alpha \beta } = \left( {\begin{array}{*{20}{c}} {P_{xx}} & {P_{xy}} & {P_{xz}} \\ {P_{yx}} & {P_{yy}} & {P_{yz}} \\ {P_{zx}} & {P_{zy}} & {P_{zz}} \end{array}} \right)} \\ 	{\hskip14pt} = {\left( {\begin{array}{*{20}{c}} { - 1} & 0 & 0 \\ { \mp \displaystyle\frac{{2\left| {{\mathbf{A}}_ \bot \times {\mathbf{B}}_ \bot } \right|}}{{A_ \bot ^2 + B_ \bot ^2}}D} & {\displaystyle\frac{{A_{ \bot y}^2 - A_{ \bot z}^2 + B_{ \bot y}^2 - B_{ \bot z}^2}}{{A_ \bot ^2 + B_ \bot ^2}}} & {2\displaystyle\frac{{A_{ \bot y}A_{ \bot z} + B_{ \bot y}B_{ \bot z}}}{{A_ \bot ^2 + B_ \bot ^2}}} \\ { \mp \displaystyle\frac{{2\left| {{\mathbf{A}}_ \bot \times {\mathbf{B}}_ \bot } \right|}}{{A_ \bot ^2 + B_ \bot ^2}}D} & {2\displaystyle\frac{{A_{ \bot y}A_{ \bot z} + B_{ \bot y}B_{ \bot z}}}{{A_ \bot ^2 + B_ \bot ^2}}} & { - \displaystyle\frac{{A_{ \bot y}^2 - A_{ \bot z}^2 + B_{ \bot y}^2 - B_{ \bot z}^2}}{{A_ \bot ^2 + B_ \bot ^2}}} \end{array}} \right)}$$

The relationship between the coordination of neutron polarization and the crystal axes is illustrated in Supplementary Figure 2. **A**_⊥_ and **B**_⊥_ are spin projection vectors along the major and minor axes on the plane perpendicular to the scattering vector, respectively. *D* is the volume faction of the spin helicity domains, *D* = |*V*_RH_ − *V*_LH_|/|*V*_RH_ + *V*_LH_|, where *V*_RH_ (*V*_LH_) is volume of the right(left)-handed domain. Therefore, *P*_*xx*_ is always −1 for a magnetic reflection. *P*_*yx*_ and *P*_*zx*_ reflect the helicity domains ratio, and *P*_*yy*_ ( = −*P*_*zz*_) and *P*_*yz*_ ( = *P*_*zy*_) reflect the magnetic structure shape projected to the plane perpendicular to the scattering vector.

**Fig. 2:**
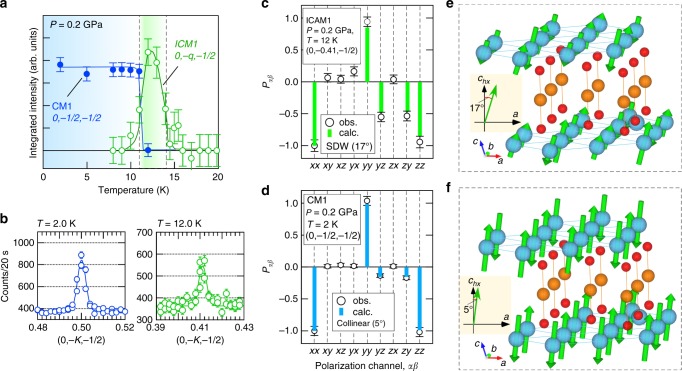
Spherical neutron polarimetry data at 0.2 GPa. **a** Temperature dependence of the integrated magnetic reflection intensities and **b** typical diffraction profiles along the [0, −*K*, −1/2] line for the spin flip channel in the *x*-direction at *P* = 0.2 GPa. Comparisons between observed and calculated polarization matrix elements for **c** the 0, −0.41, −1/2 reflection at *T* = 12 K in the ICM1 phase and **d** the 0, −1/2, -1/2 reflection at *T* = 2 K in the CM1 phase at *P* = 0.2 GPa. (The error bars in the intensity, neutron counts, and polarization matrix elements data show statistical errors.) The determined magnetic structures: **e** a collinear SDW structure with moments canted by 17 ± 2° from the *c*_*hx*_*-*axis in the *ac*-plane, and **f** a collinear commensurate structure with a canting angle of 5 ± 2°

We observed magnetic reflections at (0, −*q*, −1/2) with *q* ≃ 0.4 for 11 K ≤ *T* ≤ 14 K in the ICM1 phase, and at (0, −1/2, −1/2) below 11 K in the CM1 phase at *P* = 0.2 GPa, as is evident from the temperature dependence of the intensity and the diffraction profiles shown in Fig. 2a, b. We used the polarization channel +*x* on incident and −*x* on scattered neutrons, which represents the total magnetic scattering, for measuring the temperature dependence of integrated intensity of each magnetic Bragg reflection in all measured pressures. The quality of these data with the signal-to-noise ratio of ∼1 was sufficient to allow the determination of **P**_*αβ*_ on a reasonable time scale. Subsequently, the **P**_*αβ*_ values at *T* = 12 K and *P* = 0.2 GPa for the ICM1 phase could be compared with the calculated values for a collinear SDW structure (Fig. 2c, e). The canting angle of the spins, *φ*, from the hexagonal *c*-axis (*c*_*hx*_-axis) toward the *a*-axis in the SDW structure was found to be 17 ± 2°, which is consistent with the value of 18 ± 11° determined from a prior unpolarized neutron four-circle diffraction study^37^. (The error bars (s.d.) in the magnetic structure parameters are derived from estimation in a nonlinear least-squares fit.)

Here, we should mention magnetic domains mixing, because SNP measurements generally averaged over different magnetic domains in the sample. While CuFeO_2_ has originally rhombohedral symmetry (R$\bar 3$m) with threefold rotational symmetry above the magnetic phase transition temperature, the low-temperature magnetic ordering with *k* = (0, *q*, 1/2) (in monoclinic notation) (*k* = (*q*, *q*, 3/2) in the hexagonal notation) breaks the threefold symmetry, generating three monoclinic magnetic domains (Q-domains) with space group C2/m. The monoclinic unique axis (*b*-axis) in each Q-domain is parallel to the original [110], [1$\bar 2$0], or [$\bar 2$10] direction in the reciprocal lattice space in the hexagonal notation in R$\bar 3$m. In this case, since the magnetic reflections belonging to these three Q-domains appear at the different reciprocal positions, for example (*q*, *q*, 3/2), (*q*, −2*q*, 3/2) and (−2*q*, *q*, 3/2) in the hexagonal notation, these are not superposed to each other. We should also mention magnetic domain mixing for the canting angle *φ*. In the C2/m monoclinic space group, the canting directions, from the hexagonal *c*-axis toward the monoclinic +*a-* or −*a*-direction are not crystallographically equal to each other. In the present case, since the SDW structures with +*φ* and −*φ* give different anisotropy energy, the magnetic domain mixing for the canting angle does not exist.

In the case of the CM1 phase, the **P**_*αβ*_ matrix observed at 2 K is consistent with a collinear structure having much smaller canting moments of *φ* = 5 ± 2°, which is explained by the nonzero matrix elements, *P*_*yz*_ and *P*_*zy*_ (Fig. 2d, f). We confirmed that the canting angle in the CM1 phase remains unchanged at 2.0 GPa within the experimental accuracy, which is *φ* = 3 ± 2°, by measuring the **P**_*αβ*_ under the pressure (Supplementary Figure 3). Both collinear magnetic structures in ICM1 and CM1 phases, can be expressed by the irreducible representation (IR) in R$\bar 3$m space group, mY 1^44–46^, which confines spins in the monoclinic *ac*-plane. The magnetic point group for these phases is 2/m1′. The difference in the canting angles between ICM1 and CM1 phases can originate from the different single ion anisotropy (easy axis is along the *c*_*hx*_-axis), which varying with chemical substitution, magnetic field, or temperature^47–50^.

We also measured the full polarization matrix on the 1,1,0 or 0,0,3 nuclear reflection, to check the neutron polarizations. No neutron depolarization induced by the HAC was evident during these experiments, so these results demonstrate that SNP analysis is a feasible technique even under high pressure conditions.

### Pressure-induced phases

**Fig. 3:**
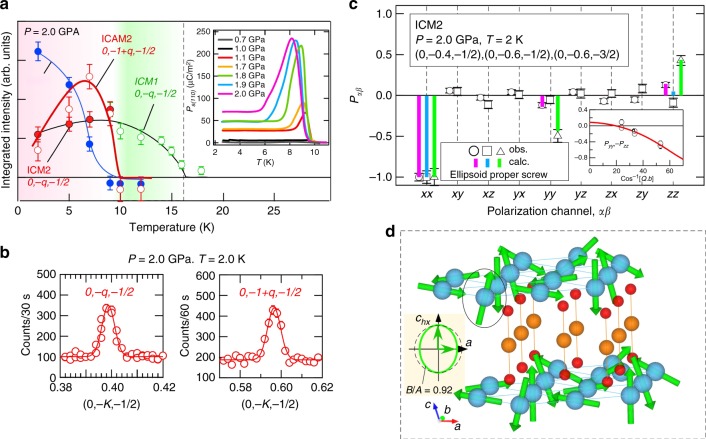
Spherical neutron polarimetry data at 2.0 GPa. **a** Temperature dependence of the integrated intensities of magnetic reflections and **b** typical diffraction profiles along the [0, −*K*, −1/2] line for the spin flip channel in the *x*-direction at *P* = 2.0 GPa. The inset in **a** shows the temperature dependence of electric polarization along the hexagonal [110]-direction (the monoclinic *b*-axis) in typical pressures up to 2.0 GPa under poling electric field 286 kV/m. **c** A comparison between observed and calculated polarization matrix elements for 0, −0.4, −1/2 (red), 0, −0.6, −1/2 (light blue), and 0, −0.6, −3/2 (light green) reflections at *T* = 2 K and *P* = 2.0 GPa. The inset in **c** shows the fitting results for the refinement of the ellipsoidal ratio |**B**|/|**A**| and the tilting angle *θ*. **d** An illustration of the magnetic structure determined under these conditions: an ellipsoidal proper screw structure with |**B**|/|**A**| = 0.92 ± 0.05

It is known that pressure-induced phase transitions occur from the CM1 phase to several incommensurate phases in CuFeO_2_^16,17^. In the previous unpolarized neutron diffraction study, we found two incommensurate magnetic orderings with *k* = (0, *q*, 1/2; *q* ≃ 0.4) for 2.5 GPa ≤ *P* ≤ 4.0 GPa in the ICM2 phase and *k* = (*q*_a_,*q*_b_,*q*_c_; *q*_a_ ≃ 0,*q*_b_ ≃ 0.34, *q*_c_ ≃ 0.42) for 4.0 GPa ≤ *P* ≤ 6.0 GPa in the ICM3 phase. As shown in Fig. 3a, b, we observed magnetic reflections corresponding to the ICM2 phase below *T* = 10.0 K at *P* = 2.0 GPa, which was coexistent with the CM1 phase below 7 K because this pressure value was close to the phase boundary. The **P**_*αβ*_ values were determined for the reflections at (0, −*q*, −1/2), (0, −1 + *q*, −1/2) and (0, −1 + *q*, -3/2), and are presented in Fig. 3c. The observed matrix elements, *P*_*yz*_ and *P*_*zy*_, were zero for all reflections measured within the experimental accuracy, which differs from the case of SDW in the ICM1 phase. Therefore, one of the ellipsoidal spiral axes (the minor axis), was parallel to the *z*-direction (the monoclinic *a*-axis), which relates to the conditions, *B*_⊥y_ = 0 and *A*_⊥z_ = 0, based on Eq. (1). The other axis (the major axis) was perpendicular to the *a*(*z*)-axis (*A*_⊥z_ = 0). We refined the direction of the major axis (the canting angle *θ* from the *c*_*hx*_-axis toward the *b*-axis) and the ellipsoidal ratio |**B**|/|**A**| for the *P*_*yy*_ and *P*_*zz*_ elements sensitive to these parameters. The refined direction of the major axis was along the *c*_*hx*_-axis, *θ* = 4 ± 4°, and the ellipsoidicity parameter was |**B**|/|**A**| = 0.92 ± 0.05. *P*_*yx*_ and *P*_*zx*_ were zero within the experimental accuracy, meaning that the helicity domains (both right-handed and left-handed) of the spiral structure were equally populated (*D* = 0 in Eq. (1)), which is consistent with no electric polarization observed under zero electric field. We thus found that the magnetic structure in the ICM2 phase is the proper screw structure with slight ellipsoidicity (Fig. 3d), similar to the structures of ferroelectric phases induced by magnetic fields and impurity-substitution^42,43,51^. The magnetic order parameter can be expressed as the superposition of two identical time-odd IRs of the R$\bar 3$m space group, mY_1 ⊕_ mY_1_ combined with real and imaginary characters^44–46^. The ellipsoidal proper screw ordering results in twofold rotation and time reversal symmetry as point group elements, but breaks the inversion and the mirror plane perpendicular to the *b*-axis. The magnetic point group in the ICM2 phase is therefore polar 21′, allowing ferroelectric polarization along the *b*-axis. In fact, we observed electric polarization along the *b*-axis, as shown in the inset to Fig. 3a. The electric polarization value at *T* = 2.0 K was nearly four times smaller than that at *T* ∼ 8 K for *P* = 2.0 GPa, which is consistent with the reduction seen in the neutron intensity of ICM2 phase below *T* ∼ 8 K (Fig. 3a). The maximum electric polarization is recovered by application of magnetic field (Supplementary Figure 4).

**Fig. 4:**
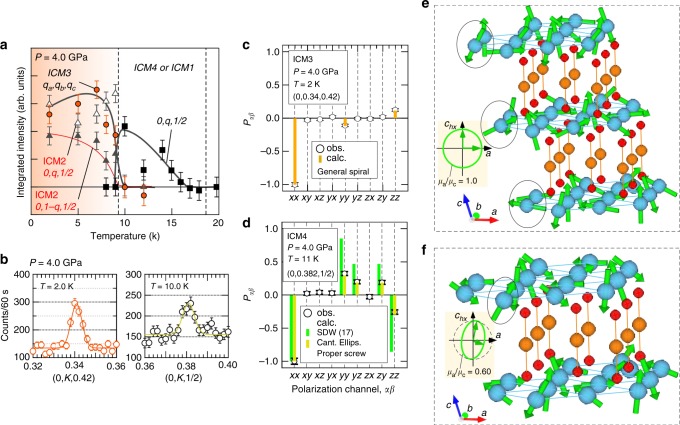
Spherical neutron polarimetry data at 4.0 GPa. **a** Temperature dependence of the integrated intensities of magnetic reflections and **b** typical diffraction profiles along the [0, *K*, 0.42] line for the ICM3 phase and the [0, *K*, 1/2] line for the ICM4 phase with the spin flip channel in the *x*-direction at *P* = 4.0 GPa. Note that the phase boundary between ICM1 and ICM4 phase at 4.0 GPa is not clear due to the sample propagation vector ***k*** = (0, *q*, 1/2), as indicated by ICM4 or ICM1. Comparisons between observed and calculated polarization matrix elements: **c** the 0, 0.34, 0.42 reflection at *T* = 2 K in the ICM3 phase and **d** the 0, 0.382, 1/2 reflection at *T* = 11 K in the ICM4 phase at *P* = 4.0 GPa, with values calculated based on SDW, using spins canted by 17° and a canted ellipsoidal proper screw model. The determined magnetic structures: **e** the general spiral structure with both cycloid modulation along the *c*-axis and proper screw modulation along the *b*-axis, and **f** the canted ellipsoidal proper screw with spins confined to the ac-plane, the major ellipsoidal axis canted by 5 ± 1°, and the ellipsoidal ratio |**B**|/|**A**| = 0.60 ± 0.02

The temperature dependence of the magnetic reflections at *P* = 4.0 GPa is shown in Fig. 4a, b. The reflection at (*q*_a_, *q*_b_, *q*_c_; *q*_a_ ≃ 0, *q*_b_ ≃ 0.34, *q*_c_ ≃ 0.42) appears below 10 K, corresponding to the ICM3 phase, together with reflections associated with the ICM2 phase because these conditions are close to the phase boundary between the ICM2 and ICM3 phases. The **k**-vector, **k** = (*q*_a_, *q*_b_, *q*_c_) with *q*_a_ = 0, *q*_b_ = 0.34, and *q*_c_ = 0.42, with general point of symmetry, for the ICM3 phase gives the two possible triclinic magnetic point groups, nonpolar $\bar 11\prime$ or polar 11′^17^. In this case, while a collinear SDW structure gives $\bar 11\prime$ a spiral structure gives 11′, as was discussed in the previous paper^17^. We have searched the spin direction of collinear SDW structure to explain these matrix element values for all directions in the 4π solid angle. However, there is no solution to explain the values for the collinear case, which proves that the magnetic point group in ICM3 phase is not the nonpolar $\bar 11\prime$ but the polar 11′. Subsequently, we searched the ellipsoid spiral structures to explain the values.

As was also the case for the ICM2 phase, both *P*_*yz*_ and *P*_*zy*_ were zero within the experimental accuracy (Fig. 4c) in ICM3 phase, meaning that one of the ellipsoidal axes was parallel to the *a*-axis. Here, assuming no ellipsoidicity a priori because of the fully ordered magnetic moments at low temperature, we determined the direction of the other axis to be nearly in the *c*_*hx*_-axis direction (*θ* = 5.9 ± 1.5°) by using the *P*_*yy*_ and *P*_*zz*_ values. Since the **k**-vector (**k** = (*q*_a_, *q*_b_, *q*_c_)) possesses an incommensurate c-component as well as a b-component in the ICM3 phase, the spiral magnetic structure is expressed by a combination of proper screw modulation along the *b*-axis and cycloid modulation along the *c*_*hx*_-direction (parallel to the monoclinic *c*^*∗*^-axis), a structure referred to as a general spiral herein (Fig. 4e). The magnetic order parameter of this general spiral structure is expressed by the superposition of the IRs R$\bar 3$m, mGP ⊕ mGP combined with real and imaginary characters^44–46^. The resultant magnetic point group is 11′, which allows ferroelectric polarization along a general direction.

**Fig. 5:**
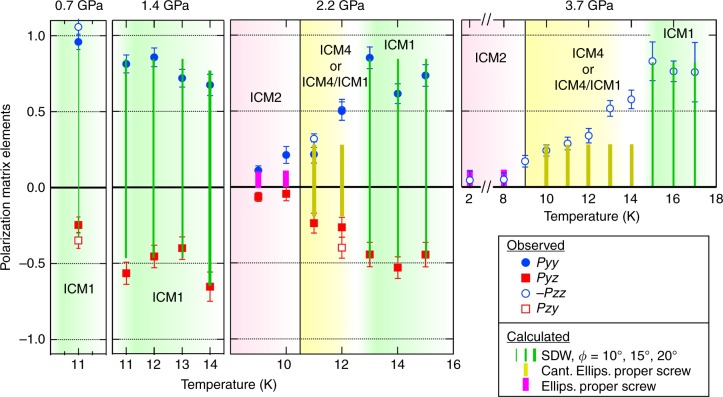
Polarization matrix elements data at several pressures. Temperature dependence of the polarization matrix elements for 0, −*q*, −1/2 reflection, *P*_*yy*_ (filled circles), *P*_*yz*_ (filled squires), −*P*_*zz*_ (open circles) and *P*_*zy*_ (open squires) at *P* = 0.7, 1.4, 2.2, and 3.7 GPa. The vertical bars represent the calculated values: the spin-density-wave structure with *φ* = 10°, 15°, 20°, the canted ellipsoidal proper screw with 0.60 ellipsoidal ratio and 5° canting angle and ellipsoidal proper screw with 0.92 ellipsoidal ratio. Note that we cannot distinguish a single ICM4 phase from coexistence of ICM4 and ICM1 phases for the intermediate temperature regions at 2.2 and 3.7 GPa in the present experiment, as indicated by ICM4 or ICM4/ICM1. The solid lines denote the phase transition temperatures between ICM2 and ICM4

In order to investigate the stability of the SDW spin ordering in the ICM1 phase at high pressure, we systematically measured the **P**_*αβ*_ matrix on **Q** = (0, *q*, 1/2) (or (0, −*q*, −1/2)) for the intermediate temperature region at 0.7, 1.4, 2.2, 3.7, and 4.0 GPa. As shown in Fig. 4d, the observed matrix elements were completely different from those obtained for the ICM1 phase: the *P*_*yz*_, *P*_*zy*_, *P*_*yy*_, and *P*_*zz*_ values at 4.0 GPa were much smaller than the values calculated using the SDW model for the ICM1 phase. A search for structural models that explain the experimental **P**_*αβ*_ at 11 K and 4.0 GPa did not identify any collinear SDW structures that fit the experimental data. The difference in the matrix elements was also seen in lower pressure region. The temperature dependence of *P*_*yy*_ (−*P*_*zz*_) and *P*_*yz*_ (*P*_*yz*_), sensitive to magnetic structure model, for 0.7 GPa ≤ *P* ≤ 3.7 GPa is summarized in Fig. 5. The *P*_*yy*_ and *P*_*yz*_ observed at 0.7 and 1.4 GPa can be explained by the SDW model for the data measured at *T* ≥ 11 K. These results are consistent with the absent macroscopic electric polarization above 10 K up to 2.0 GPa (the inset of Fig. 3a). In contrast, for the data at 2.2 GPa and 3.7 GPa, the *P*_*yy*_ (−*P*_*zz*_) and *P*_*yz*_ (*P*_*zy*_) values deviate from those of SDW model below *T* ∼ 12 and *T* ∼ 13.5 K, respectively. These results indicate that a phase transition occurs from the ICM1 phase in low-pressure region to another phase in high-pressure region, which is defined as ICM4 phase (Fig. 1b). The super lattice reflection at **Q** = (0, 1 − *q*, 1/2), characteristic of the ICM2 phase, was not observed in the ICM4 phase, which indicates that the low-temperature ICM2 phase does not coexist with ICM4 phase. However, in the present experiment, we cannot distinguish the two possibilities, either phase mixing with the ICM1 phase inside the ICM4 phase, or that a single ICM4 phase exists, due to the same peak position at **Q** = (0, *q*, 1/2) (or **Q** = (0, −*q*, −1/2)). The gradual change in the matrix elements with varying temperature at 2.2 and 3.7 GPa in ICM4 phase (Fig. 5) can be caused by either change in the volume fraction between ICM1 and ICM4 phases or a continuous changing of the spin noncollinearity toward the collinear SDW (with warming), as discussed shortly.

Here, we investigate the magnetic structure model in the ICM4 phase by assuming that the ICM4 phase is realized as a single phase at 11 K and 4.0 GPa. As shown in Fig. 4d, f, we found one possible structure model with monoclinic magnetic symmetry. That is an ellipsoidal proper screw with spins on the *ac*-plane and the major axis tilted by 5 ± 1° from the *c*_*hx*_-axis toward the *a*-axis (to which the minor axis is perpendicular) and a 0.60 ± 0.02 ellipsoidal ratio (Fig. 4d, f), which gives the monoclinic magnetic point group 21′. The determined large ellipsoidal ratio might be caused by the fact that the magnetic moments are not fully ordered due to the relatively high temperature compared to the phase transition temperature. We here neglected the spin bunching in the ellipsoidal structure. Although we need to measure reflections of the higher order harmonics, it is difficult to measure the reflections, given as much smaller intensities. The ellipsoidal proper screw model can explain the experimental data for 11 K at 2.2 GPa, from 10 to 12 K at 3.7 K as well as 11 K at 4.0 GPa, as shown in Fig. 5. However, the data measured close to the phase boundary between ICM1 and ICM4 phases, 12 K at 2.2 GPa, 13 K and 14 K at 3.7 GPa, do not fit the calculated value. The difference in observed and calculated data might be caused either by that the magnetic structure parameters, ellipsoidal ratio and canting angle, are changed gradually or by that phase mixing contribution of ICM1 phase is varying in this temperature and pressure region. It should be also noted that there is another model with triclinic symmetry an ellipsoidal spiral with spins along a general direction that also explains the experimental data. Consequently, from the present experiment, we have presented one possible magnetic structure for the ICM4 phase, which is the canted ellipsoidal proper screw structure, by assuming the existence of a single magnetic phase, no spin bunching exists, and keeping monoclinic symmetry. For further understanding the ICM4 phase, macroscopic polarization measurements under high pressure as well as the SNP experiment are needed.

The magnetic order parameter of the canted ellipsoidal proper screw ordering in ICM4 phase can be expressed as the superposition of the IRs R$\bar 3$m, mY_1 ⊕_ mY_1_ combined with real and imaginary characters, which leads to the magnetic point group 21′, allowing polarization along the *b*-axis. As mentioned above, the SDW structure in ICM1 phase belongs to the 2/m1′ magnetic point group, including the symmetry operations, twofold rotation, mirror, time reversal and inversion. At the phase transition, the canted ellipsoidal proper screw structure in ICM4 phase leaves only twofold rotation and time reversal symmetry as point group elements, but breaks mirror plane and inversion symmetry, leading to the magnetic point group 21′ in ICM4 phase. The phase transition can be explained as the emergence of the additional spin component approximately perpendicular to the collinear spin component of the SDW structure. This kind of phase transition also happens in other Heisenberg triangular-lattice antiferromagnets with weak easy axis anisotropy, such as CsNiCl_3_^52^ and CuCrO_2_^53^.

## Discussion

Here, it is helpful to discuss the origins of the observed and expected ferroelectric polarizations in the pressure-induced phases ICM2, ICM3, and ICM4 in CuFeO_2_. For the ICM2 and ICM4 phases with *k* = (0, *q*, 1/2), the magnetic structure has only a proper screw component in which the spiral spins are confined to the *ac*-plane perpendicular to the modulation direction, the *b*-axis. The magnetic point group in these phases is 21′, allowing ferroelectric polarization along the *b*-axis (the twofold axis), which is consistent with the observed polarization in the ICM2 phase. The inverse Dzyaloshinskii–Moriya (DM) effect^54,55^ and spin–current mechanism^56^, which are represented by **p** ∝ **r**_**ij**_ × (**S**_**i**_ × **S**_**j**_)(≡ **p**_1_), are not applicable to the proper screw orderings in the ICM2 and ICM4 phases, due to **r**_**ij**_ ||(**S**_**i**_ × **S**_**j**_). When a crystal has neither a mirror plane containing **r**_**ij**_ nor an *n*-fold rotation axis perpendicular to **r**_**ij**_ (meaning that the point group belongs to the ferroaxial class^57,58^), the electric polarization can be expected to be parallel to the cross-product, **p** ∝ **S**_**i**_ × **S**_**j**_ (≡ **p**_2_), via the inverse DM effect, as proposed by Kaplan and Mahanti^59^. In fact, in some multiferroics, such as RbFe(MoO_4_)_2_ and CuNb_2_O_8_^57,60–62^, proper screw ordering generates an electric polarization parallel to **S**_**i**_ × **S**_**j**_. The electric polarization in these materials has been explained as the result of coupling between the spin chirality, **r**_**ij**_ (**S**_**i**_ × **S**_**j**_), and ferroaxial distortions, **A**, based on the inverse DM effect^57,61,62^. In CuFeO_2_, however, the room temperature space group, nonferroaxial R$\bar 3$m with threefold rotational symmetry parallel to the hexagonal *c*-axis, does not satisfy the symmetry condition allowing the **p**_2_ component. Nevertheless, the crystal is distorted to monoclinic ferroaxial C2/m below the magnetic ordering temperature^29–32^. Therefore, the observed electric polarization in the ICM2 phase, which is parallel to the *b*-axis (||**A**), can be explained by coupling between the spin helicity and the ferroaxial distortion induced as a secondary order parameter in CuFeO_2_. For the ICM3 phase, the general spiral structure possesses both proper screw modulation along the *b*-axis and cycloid modulation along the *c*^*∗*^-direction, leading to the triclinic magnetic point group, 11′, which allows electric polarization along a general direction. Considering the inverse DM mechanism described above, we can expect that the proper screw component generates polarization along the *b*-axis as the **p**_2_ component, while the cycloid modulation induces polarization along the *a*-axis as the **p**_1_ component in the ICM3 phase. However, testing this theory will require polarization measurements at higher pressures.

To understand the numerous pressure-induced phase transitions exhibited by CuFeO_2_, it is helpful to discuss the dominant exchange interactions affected by pressure. The strongest exchange interaction in this system is the nearest neighbor antiferromagnetic interaction in the triangular-lattice plane, *J*_1_^47^. As reported by Mekata et al.^63^, *J*_1_ is the sum of the direct Fe–Fe exchange interaction, which is assumed to be ferromagnetic, and the antiferromagnetic Fe–O–Fe superexchange interaction through O^2−^ ions. The 180° superexchange interaction in a 3d^5^ system is antiferromagnetic^64^, and is expected to be stronger than the 90° interaction. Since the Fe–O–Fe angle is ∼97° in CuFeO_2_, the superexchange interaction can be determined from the superposition of the strong antiferromagnetic 180° and the weaker 90° interactions. The application of hydrostatic pressure shortens the Fe–Fe bond length while decreasing the Fe–O–Fe bond angle, as demonstrated by previous X-ray diffraction experiments^65^. Since the ferromagnetic direct exchange interaction increases and the antiferromagnetic superexchange interaction decreases under pressure, the antiferromagnetic interaction *J*_1_ can be reduced by pressure. Conversely, although the inter-plane exchange interaction (defined as the superexchange interaction through the exchange path Fe–O–Cu–O–Fe) is also antiferromagnetic, the application of pressure is believed to increase the antiferromagnetic interaction. The antiparallel inter-plane spin configuration contracts the *c*-axis below the Néel temperature to reduce the exchange energy, and the resulting shorter distances correspond to a stronger antiferromagnetic interaction^29–32^. In contrast, the antiferromagnetic ordering in the triangular-lattice plane elongates the *b*-axis because this elongation decreases the ferromagnetic direct exchange and increases the antiferromagnetic superexchange interactions. We can thus argue that the anisotropic changes in the exchange interactions resulting from the application of pressure affect the delicate balance of the competing intra-layer and inter-layer interactions, and produce the rich magnetoelectric phase diagram of CuFeO_2_. In fact, when the nonmagnetic Cu site is substituted with Ag ions, leading to anisotropic changes in the delafossite lattice, a different incommensurate ordering (cycloid) is stabilized in AgFeO_2_^51,66^.

We should also mention the limitation of the SNP experiment under pressure with using the HAC. Considering the data accuracy of the observed polarization matrix elements at 4.0 GPa in the present SNP experiment, the present case, 0.04 mm^3^ (0.4 × 0.5 × 0.2 mm^3^), and ∼0.09 μ_B_Å^−3^, can be considered as close to the limit of feasibility. Osakabe et al. have succeeded in pressurizing the different sample with almost the same sample volume as the present case up to 9.8 GPa by using the HAC^15^. Therefore, basically one can reach such the high pressure with the present setup. Furthermore, when incident neutron flux significantly increases from the present case, 1 × 10^7^ n cm^−2^ s^−1^, in future, we will be able to increase the maximum pressure by reducing the sample volume. We should also mention a disadvantage of the SNP experiment under pressure. Since higher harmonic components generally give significantly small intensity of the magnetic Bragg reflections, it is difficult to determine the magnetic structure model including spin bunching in SNP experiment under pressure.

In conclusion, we have succeeded in carrying out the first SNP experiments in conjunction with the application of pressure, working with the multiferroic delafossite CuFeO_2_ and using the newly developed nonmagnetic HAC. This work determined the detailed magnetic structures in the pressure-induced ferroelectric phases of this material, as well as nonpolar phases at ambient pressure. We confirmed the magnetic structures in the ICM1 and CM1 phases to be collinear structures, and found precise spin canting angles of 17 ± 2° and 5 ± 2° for the ICM1 and CM phases, respectively. In the case of the ICM2 phase, an ellipsoidal proper screw structure with the 21′ magnetic point group was determined, with an ellipsoidicity ratio of 0.92 ± 0.05. This magnetic symmetry is consistent with the observed electric polarization. We also elucidated the magnetic structure in the ICM3 phase, and found a spiral structure with spins confined to the *ac*-plane. Since the **k**-vector is of triclinic symmetry *k* = (0, 0.34, 0.42), this magnetic structure (termed the general spiral) possesses both a cycloidal modulations along the *c*-axis and proper screw modulations along the *b*-axis in the ICM3 phase, which has the 11′ point group, allowing electric polarization along a general direction. This study also identified the existence of the phase transition between the ICM1 and ICM4 phases in the intermediate temperature region. One possible magnetic structure in the ICM4 phase is presented to be a canted ellipsoidal proper screw with the 21′ point group. Finally, the present study provides evidence that SNP measurements are viable even in combination with high-pressure conditions. It is our hope that the present technique will allow researchers to elucidate pressure-induced physical phenomena associated with complex magnetic ordering.

## Methods

### Neutron polarimetry analysis experiment

The neutron polarimetry experiments were carried out using a CRYOPAD apparatus^12,13^ on the D3 beam line at the Institute Laue Langevin (ILL). Single-crystal samples of CuFeO_2_, grown by the floating zone technique, were cut into rectangular shapes with dimensions of 1.1 × 1.1 × 0.2 mm^3^, 0.5 × 0.5 × 0.2 mm^3^, and 0.4 × 0.5 × 0.2 mm^3^ for experiments at *P* = 0.2 and 2.0 GPa, *P* = 0.7, 1.4, 2.2, and 3.7 GPa, and *P* = 4.0 GPa, respectively. The crystals have mosaic widths, 0.40 ± 0.02, 0.31 ± 0.04, and 0.50 ± 0.05, respectively. The crystal qualities were kept even under pressure up to 4.0 GPa, by using glycerin as the pressure transmission medium that can be used up to ∼5.5 GPa without serious pressure uniaxiality^67,68^. The cut samples were mounted in the HAC with the monoclinic *a*-axis (hexagonal [1$\bar 1$0]) vertical, in order to provide access to the monoclinic (0, *K*, *L*) (hexagonal (*H*, *H*, *L*)) reflections. The incident neutrons are polarized and monochromatized at the Heusler monochrometer. The incident wavelength 0.85 Å was employed. The final neutron spin was analyzed with ^3^He filter cells. The data has been corrected for the exponential decay of the ^3^He polarization. The polarization matrices were calculated with the Mag2Pol program^69^.

### Hybrid anvil cell

A sapphire anvil with culet sizes, 4.2, 2.7, or 2.4 mm diameter, supported by CuBe or MP35N alloys were used as an anvil on one side. For the opposite side, a nonmagnetic diamond composite (with a SiC binder) and a WC with a nonmagnetic Ni binder were exployed up to 2.0 and 4.0 GPa, respectively. These materials were confirmed to be paramagnetic at *T* = 2 K by magnetization measurements (Supplementary Figure 1). Aluminum gaskets (Al2017) with 2.0 or 1.0 mm diameter hole were used for the experiment for *P* = 0.2 and 2.0 GPa, and *P* = 0.7, 1.4, 2.2, 3.7, and 4.0 GPa, respectively. The HAC was inserted into a standard He cryostat (ILL Orange). Pressures were determined based on the known transition temperatures of CuFeO_2_^17^. The accuracy of pressure was estimated to be ±0.25 GPa.

### Pyroelectric current measurement

For the pyroelectric current measurements, the single crystal in the glycerin pressure medium was pressurized in a clamp cell (HPC-32, ElectroLAB Company). The sample dimension for the measurements was 0.4 × 2.0 × 0.7 (thickness) mm^3^. A Keithley 6517B electrometer was employed.

## Electronic supplementary material

Supplementary Information

## Data Availability

The data that support the plots within this paper and other findings of this study are available from the corresponding author upon reasonable request.
