# Supplementary material for: Spherical neutron polarimetry under high pressure for a multiferroic delafossite ferrite
Source: Nat Commun. 2018 Oct 22;9:4368. doi: 10.1038/s41467-018-06737-6 (PMC6197275; doi:10.1038/s41467-018-06737-6)
Supplement: Supplementary file 1 — Supplementary Information [file 41467_2018_6737_MOESM1_ESM.pdf]

Spherical Neutron Polarimetry under High-Pressure for Multiferroic Delafossite Ferrite

Terada et al.

I. SUPPLEMENTARY NOTE 1. MAGNETIZATION CURVES OF ANVILS

In order to develop nonmagnetic Hybrid-Anvil-Cell (HAC), we checked magnetizations of the anvils and body of the cell, which were used in the spherical neutron polarimetry (SNP) analysis experiment. At room temperature, the diamond composite with SiC-binder, and CuBe alloy exhibit diamagnetic behavior, while the WC with Ni-binder and MP35N alloy show paramagnetic. (Fig. Supplementary Figure 1) At low temperature, T = 2.0 K, all the materials show paramagnetic magnetization curve. In the SNP experiment with the HAC, we did not see any neutron depolarization.

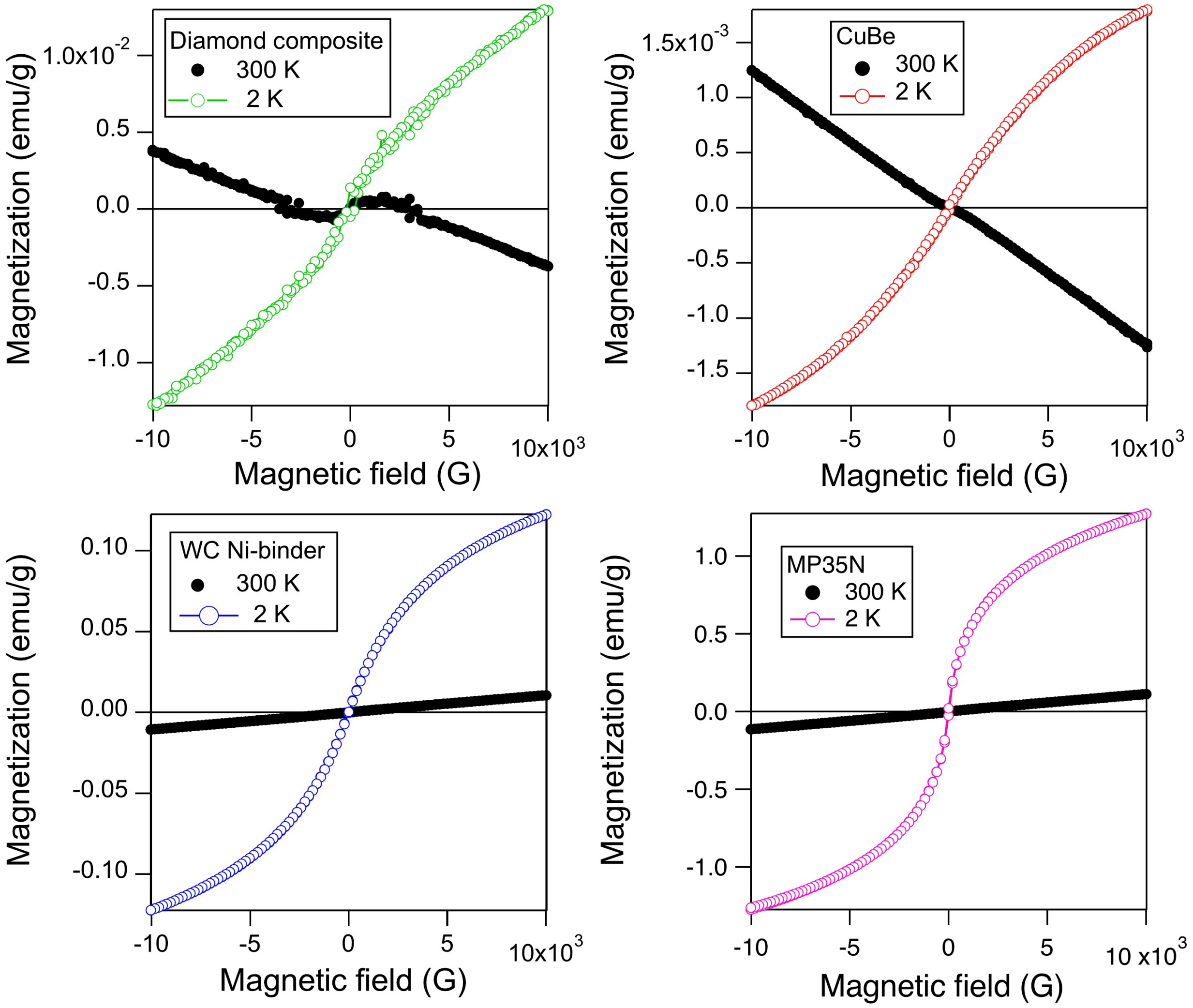

Supplementary Figure 1: Magnetization curve at room temperature and T = 2.0 K for diamond composite with SiC-binder, WC with Ni-binder, CuBe alloy and MP35N alloy.

II. SUPPLEMENTARY NOTE 2. DERIVATION OF $P_{\alpha\beta}$ MATRIX

A. Cross-section and polarization of scattered neutrons

An ellipsoidal spiral is defined as

$$\begin{aligned} \boldsymbol{S}_l &= \langle S_{\mathrm{long}}\rangle \cos(\boldsymbol{k}\cdot\boldsymbol{l}) - \langle S_{\mathrm{short}}\rangle \sin(\boldsymbol{k}\cdot\boldsymbol{l}) \\ &= (\boldsymbol{A}+i\boldsymbol{B})\exp(i\boldsymbol{k}\cdot\boldsymbol{l}) + (\boldsymbol{A}-i\boldsymbol{B})\exp(-i\boldsymbol{k}\cdot\boldsymbol{l}), \end{aligned} \tag{1}$$

where $|\boldsymbol{A}| = \langle S_{\mathrm{long}}\rangle/2$ and $|\boldsymbol{B}| = \langle S_{\mathrm{short}}\rangle/2$.

Scattering cross-section for polarized neutron is expressed as,[1, 2]

$$\frac{d\sigma}{d\Omega} = \boldsymbol{M}_\perp(\boldsymbol{Q})\cdot\boldsymbol{M}_\perp(\boldsymbol{Q})^* + i\boldsymbol{P}\cdot[\boldsymbol{M}_\perp(\boldsymbol{Q})^*\times\boldsymbol{M}_\perp(\boldsymbol{Q})], \tag{2}$$

where $\boldsymbol{Q}$ is scattering vector,

$$\begin{aligned} \boldsymbol{M}_\perp(\boldsymbol{Q}) &= \boldsymbol{M}(\boldsymbol{Q}) - |\boldsymbol{M}_\perp(\boldsymbol{Q})\cdot\boldsymbol{Q}|\hat{\boldsymbol{Q}}, \\ \boldsymbol{M}(\boldsymbol{Q}) &= \gamma r_0 \sum_l f(\boldsymbol{Q})\boldsymbol{S}_l \exp(i\boldsymbol{Q}\cdot\boldsymbol{l}), \end{aligned}$$

where γ is constant, r_0 is classical electronic radius, $f(\boldsymbol{Q})$ is magnetic form factor, and $\boldsymbol{l}$ is position vector in lattice.

From the equations (1) and (2), we obtain

$$\begin{aligned} \boldsymbol{M}_\perp(\boldsymbol{Q}) &= \gamma r_0 f(\boldsymbol{Q})(\boldsymbol{A}_\perp + i\boldsymbol{B}_\perp)\sum_l \exp(i(\boldsymbol{Q}+\boldsymbol{k})\cdot\boldsymbol{l})) \\ &+ \gamma r_0 f(\boldsymbol{Q})(\boldsymbol{A}_\perp + i\boldsymbol{B}_\perp)^*\sum_l \exp(-i(\boldsymbol{Q}-\boldsymbol{k})\cdot\boldsymbol{l})), \end{aligned} \tag{3}$$

where $\boldsymbol{A}_\perp$ and $\boldsymbol{B}_\perp$ are projection vectors of $\boldsymbol{A}$ and $\boldsymbol{B}$ to the plane perpendicular to $\boldsymbol{Q}$.

Substituting equation (3) for equation (2), we obtain the following equation for Bragg conditions,

$$\left(\frac{d\sigma}{d\Omega}\right)_{\boldsymbol{Q}=\boldsymbol{\tau}\pm\boldsymbol{k}} = (\gamma r_0 f(\boldsymbol{Q}))^2\left[|\boldsymbol{A}_\perp|^2 + |\boldsymbol{B}_\perp|^2 \pm 2\boldsymbol{P}\cdot\boldsymbol{Q}|\boldsymbol{A}_\perp\times\boldsymbol{B}_\perp|D\right], \tag{4}$$

where D is volume fraction of helicity domains (right-handed (V_{RH}) or left handed (V_{LH})),

$$D = \frac{V_{RH}-V_{LH}}{V_{RH}+V_{LH}},$$

and we define the left-handed helicity so that $(\boldsymbol{A}_\perp\times\boldsymbol{B}_\perp)||\boldsymbol{Q}$.

The scattering cross-section including scattered neutron polarization, $\boldsymbol{P}_f$, is expressed as,[1]

$$\begin{aligned} \boldsymbol{P}_f\left(\frac{d\sigma}{d\Omega}\right) &= \big(\boldsymbol{M}_\perp(\boldsymbol{Q})^*\cdot\boldsymbol{P})\boldsymbol{M}_.(\boldsymbol{Q})\big) + \big(\boldsymbol{M}_\perp(\boldsymbol{Q})\cdot\boldsymbol{P}\big)\boldsymbol{M}_.(\boldsymbol{Q})^* \\ &- \big(\boldsymbol{M}_\perp(\boldsymbol{Q})\cdot\boldsymbol{M}_\perp(\boldsymbol{Q})^*\big)\boldsymbol{P} - i\big(\boldsymbol{M}_\perp(\boldsymbol{Q})^*\times(\boldsymbol{M}_\perp(\boldsymbol{Q})^*\big) \end{aligned} \tag{5}$$

Substituting equation (3) for equation (5), we obtain the following equation for Bragg conditions,

$$\begin{aligned} \boldsymbol{P}_f\left(\frac{d\sigma}{d\Omega}\right)_{\boldsymbol{Q}=\boldsymbol{\tau}\pm\boldsymbol{k}} &= 2(\gamma r_0 f(\boldsymbol{Q}))^2\Big[(\boldsymbol{A}_\perp\cdot\boldsymbol{P})\boldsymbol{A}_\perp + (\boldsymbol{B}_\perp\cdot\boldsymbol{P})\boldsymbol{B}_\perp \\ &- \big(|\boldsymbol{A}_\perp|^2 + |\boldsymbol{B}_\perp|^2\big)\boldsymbol{P} \mp |\boldsymbol{A}_\perp\times\boldsymbol{B}_\perp|D\boldsymbol{P}\Big] \end{aligned} \tag{6}$$

Here, we obtain polarization of scattered neutrons by equations (4) and (6).

B. Matrix elements of $\boldsymbol{P}_{\alpha\beta}$

$P_{\alpha\beta}$ is defined to be a polarization ratio of scattered neutrons along β $(= x, y, z)$ direction when incident neutron vector is parallel to α $(= x, y, z)$ direction. x-axis is parallel to scattering vector $\boldsymbol{Q}$, z-axis is perpendicular to the scattering plane, and y-axis is orthogonal to x- and z-axes in right-handed Certesian coordination.

1. $\boldsymbol{P}||x$

When the incident neutron polarization is parallel to x,

$$(\boldsymbol{A}_\perp \cdot \boldsymbol{P}) = (\boldsymbol{B}_\perp \cdot \boldsymbol{P}) = 0. \tag{7}$$

Therefore, using equations (4) and (6), we obtain the polarization ratio

$$\boldsymbol{P_f} = \frac{-(|\boldsymbol{A}_\perp|^2 + |\boldsymbol{B}_\perp|^2)\boldsymbol{P} \mp 2|\boldsymbol{A}_\perp \times \boldsymbol{B}_\perp| D\boldsymbol{P}}{|\boldsymbol{A}_\perp|^2 + |\boldsymbol{B}_\perp|^2 \pm 2|\boldsymbol{A}_\perp \times \boldsymbol{B}_\perp| D}, \tag{8}$$

which leads to

$$P_{xx} = -1 \tag{9}$$
$$P_{xy} = 0 \tag{10}$$
$$P_{xz} = 0. \tag{11}$$

2. $\boldsymbol{P}||y$

When the incident polarization parallel to y,

$$\boldsymbol{P} = \begin{pmatrix} 0 \\ 1 \\ 0 \end{pmatrix}, \boldsymbol{A}_\perp = \begin{pmatrix} 0 \\ A^y_\perp \\ A^z_\perp \end{pmatrix}, \boldsymbol{B}_\perp = \begin{pmatrix} 0 \\ B^y_\perp \\ B^z_\perp \end{pmatrix} \tag{12}$$

Substituting them for equations (4) and (6), we obtain

$$\left(\frac{d\sigma}{d\Omega}\right)_{\boldsymbol{Q}=\boldsymbol{\tau}\pm\boldsymbol{k}} = (\gamma r_0 f(\boldsymbol{Q}))^2 (|\boldsymbol{A}_\perp|^2 + |\boldsymbol{B}_\perp|^2) \tag{13}$$

$$\boldsymbol{P_f}\left(\frac{d\sigma}{d\Omega}\right)_{\boldsymbol{Q}=\boldsymbol{\tau}\pm\boldsymbol{k}} = (\gamma r_0 f(\boldsymbol{Q}))^2 \big[2(A^y_\perp \boldsymbol{A}_\perp + B^y_\perp \boldsymbol{B}_\perp) - (|\boldsymbol{A}_\perp|^2 + |\boldsymbol{B}_\perp|^2)\boldsymbol{P} \mp 2|\boldsymbol{A}_\perp \times \boldsymbol{B}_\perp| D\boldsymbol{P}\big]. \tag{14}$$

Using equations (13) and (14), we obtain

$$P_{yx} = \mp \frac{2|\boldsymbol{A}_\perp \times \boldsymbol{B}_\perp| D}{|\boldsymbol{A}_\perp|^2 + |\boldsymbol{B}_\perp|^2} \tag{15}$$
$$P_{yy} = \frac{2(A^y_\perp)^2 + 2(B^y_\perp)^2 - (|\boldsymbol{A}_\perp|^2 + |\boldsymbol{B}_\perp|^2)}{|\boldsymbol{A}_\perp|^2 + |\boldsymbol{B}_\perp|^2} \tag{16}$$
$$P_{yz} = \frac{2A^y_\perp A^z_\perp + 2B^y_\perp B^z_\perp}{|\boldsymbol{A}_\perp|^2 + |\boldsymbol{B}_\perp|^2}. \tag{17}$$

3. $\boldsymbol{P}||z$

We can obtain the matrix elements in the same way as $\boldsymbol{P}||y$ case as follows.

$$P_{zx} = \mp \frac{2|\boldsymbol{A}_\perp \times \boldsymbol{B}_\perp| D}{|\boldsymbol{A}_\perp|^2 + |\boldsymbol{B}_\perp|^2} \tag{18}$$
$$P_{zy} = \frac{2A^y_\perp A^z_\perp + 2B^y_\perp B^z_\perp}{|\boldsymbol{A}_\perp|^2 + |\boldsymbol{B}_\perp|^2} \tag{19}$$
$$P_{zz} = \frac{2(A^z_\perp)^2 + 2(B^z_\perp)^2 - (|\boldsymbol{A}_\perp|^2 + |\boldsymbol{B}_\perp|^2)}{|\boldsymbol{A}_\perp|^2 + |\boldsymbol{B}_\perp|^2}. \tag{20}$$

Summarizing the equations (9-11), (15-17), and (18-20), we obtain the full matrix.

$$\boldsymbol{P}_{\alpha\beta} = \begin{pmatrix} P_{xx} & P_{xy} & P_{xz} \\ P_{yx} & P_{yy} & P_{yz} \\ P_{zx} & P_{zy} & P_{zz} \end{pmatrix} = \begin{pmatrix} -1 & 0 & 0 \\ \mp\frac{2|\boldsymbol{A}_\perp \times \boldsymbol{B}_\perp|}{A^2_\perp + B^2_\perp} D & \frac{A^2_{\perp y} - A^2_{\perp z} + B^2_{\perp y} - B^2_{\perp z}}{A^2_\perp + B^2_\perp} & 2\frac{A_{\perp y}A_{\perp z} + B_{\perp y}B_{\perp z}}{A^2_\perp + B^2_\perp} \\ \mp\frac{2|\boldsymbol{A}_\perp \times \boldsymbol{B}_\perp|}{A^2_\perp + B^2_\perp} D & 2\frac{A_{\perp y}A_{\perp z} + B_{\perp y}B_{\perp z}}{A^2_\perp + B^2_\perp} & -\frac{A^2_{\perp y} - A^2_{\perp z} + B^2_{\perp y} - B^2_{\perp z}}{A^2_\perp + B^2_\perp} \end{pmatrix}, \tag{21}$$

III. SUPPLEMENTARY NOTE 3. RELATIONSHIP BETWEEN COORDINATION OF NEUTRON POLARIZATION AND CRYSTAL

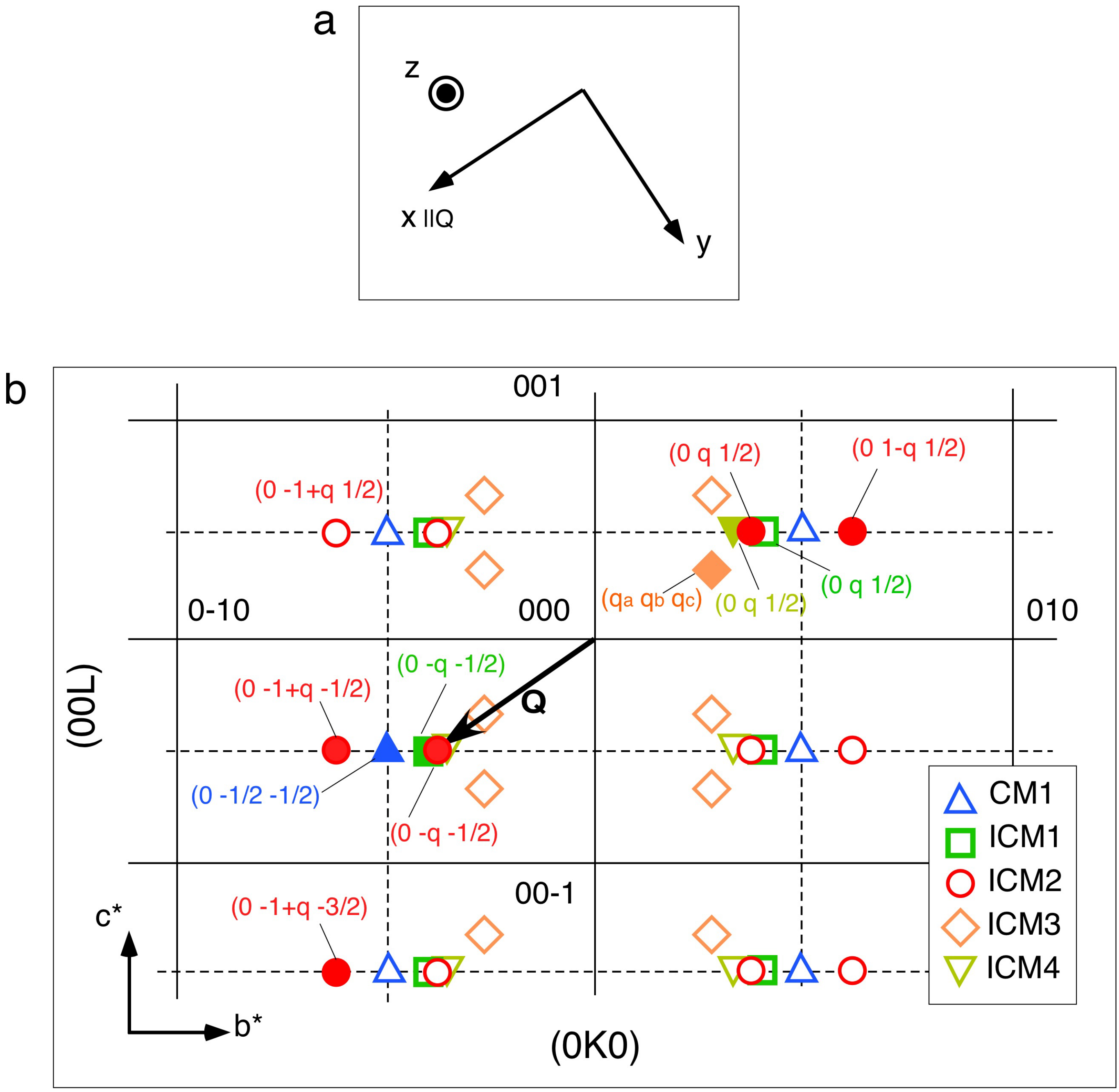

Supplementary Figure 2: Relationship between coordination of neutron polarization for $\boldsymbol{Q} = (0, -q, -\frac{1}{2})$ and the reciprocal lattice $(0, K, L)$ zone. Triangle, square, circle, diamond and inverted triangle symbols denote the reflection positions for CM1, ICM1, ICM2, ICM3 and ICM4 phases, respectively. Filled symbols denote reflections observed in each pressure as follows; $(0, -1/2, -1/2)$ in CM1 phase at $P = 0.2$ and 2.0 GPa, $(0, -q, -1/2)$ in ICM1 phase at $P = 0.2$, 0.7, 1.4, 2.2 and 3.7 GPa, $(0, -q, -1/2)$ in ICM4 at P =2.2 and 3.7 GPa, $(0, -q, -1/2)$, $(0, -1+q, -1/2)$ and $(0, -1+q, -3/2)$ in ICM2 phase at $P = 2.0$ GPa, $(0, q, 1/2)$ and $(0, 1-q, 1/2)$ in ICM2 phase at $P = 4.0$ GPa, (q_a, q_b, q_c) in ICM3 at P =4.0 GPa, and $(0, q, 1/2)$ in ICM4 phase at P =4.0 GPa.

IV. SUPPLEMENTARY NOTE 4. FULL POLARIZATION MATRIX OBSERVED AT 2.0 GPA FOR CM1 PHASE

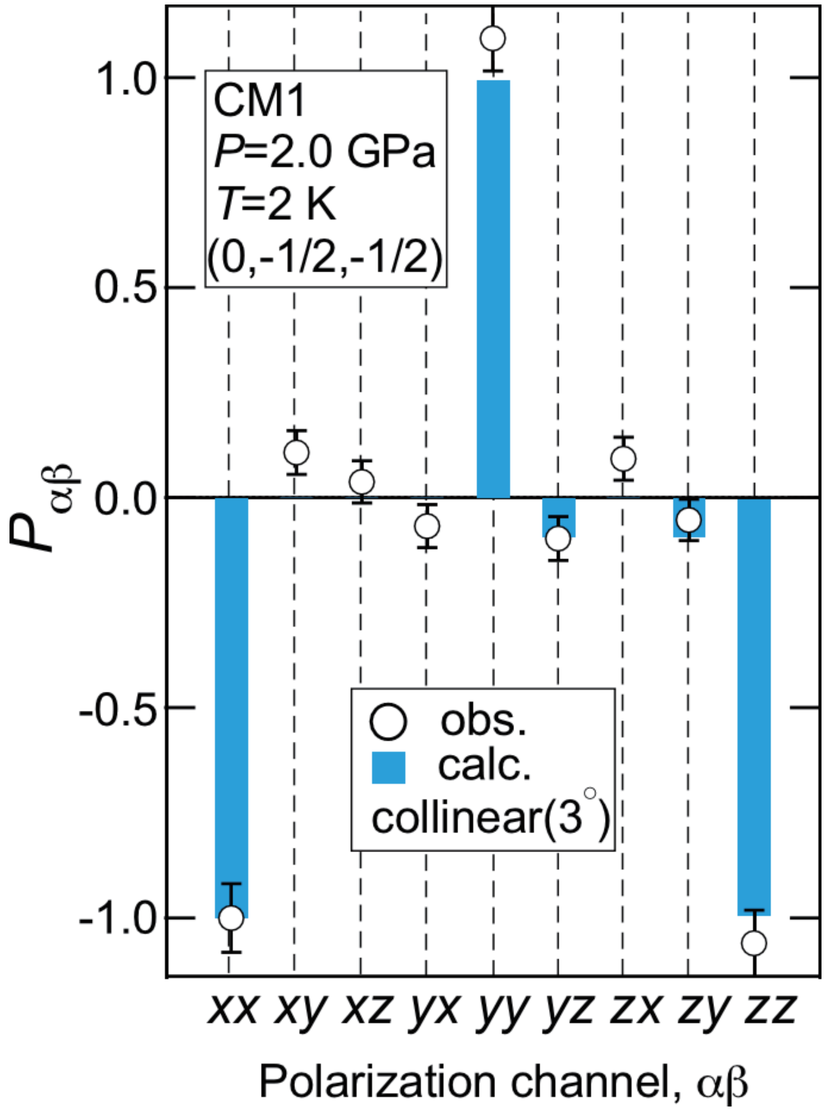

Supplementary Figure 3: Comparison between the observed and calculated full polarization matrix observed at $(0, -\frac{1}{2}, -\frac{1}{2})$ for the CM1 phase. The collinear structure with the canting angle $3 \pm 2^{\circ}$ explains the experimental data, which is the same value as the angle at 0.2 GPa $(5 \pm 2^{\circ})$ within the experimental accuracy.

V. SUPPLEMENTARY NOTE 5. MAGNETIC FIELD DEPENDENCE OF FERROELECTRIC POLARIZATION AT $P = 1.9$ GPA

Figure S4 shows temperature dependence of electric polarization in the hexagonal ab-plane at P =1.9 GPa and typical magnetic fields in $CuFeO_2$. These data were measured under poling electric field 286 kV/m.

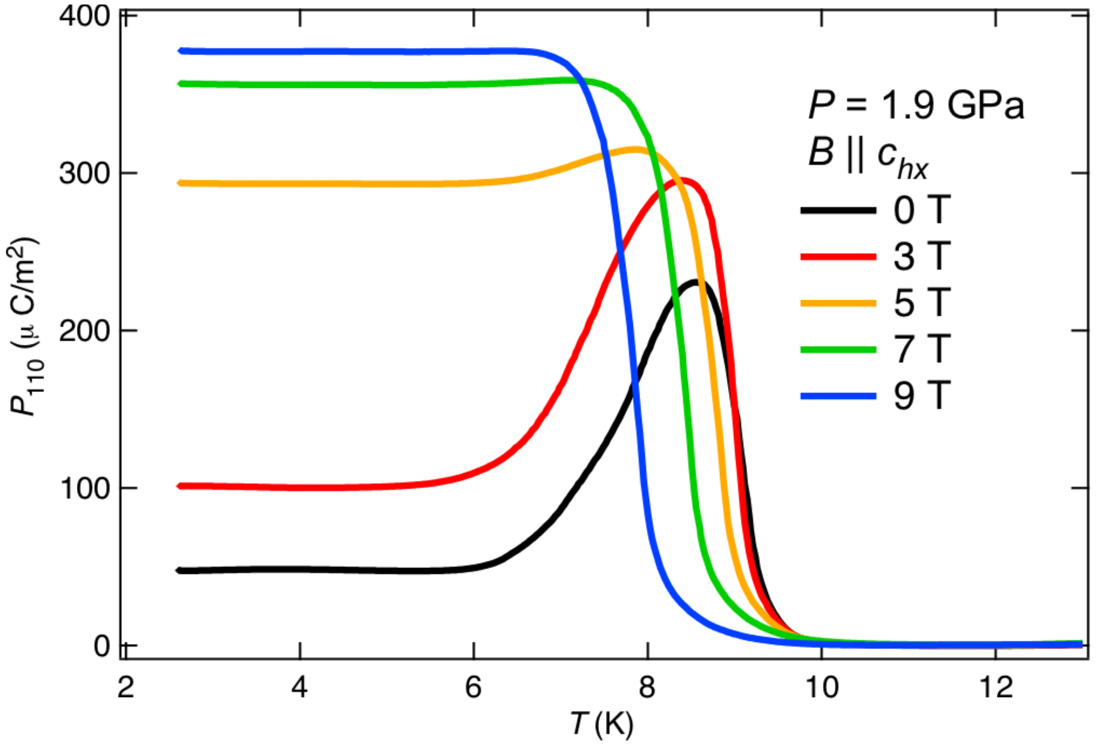

Supplementary Figure 4: Temperature dependence of electric polarization at typical magnetic fields, observed after cooling under electric field $E = 286$ kV/m.

Supplementary References

[1] Blume, M, Phys. Rev. **130**, 1670 (1963).
[2] Nakajima, T, PhD thesis, Tokyo University of Science, March 2010.
